# Elevated CO_2_ alters distribution of nodal leaf area and enhances nitrogen uptake contributing to yield increase of soybean cultivars grown in Mollisols

**DOI:** 10.1371/journal.pone.0176688

**Published:** 2017-05-01

**Authors:** Jian Jin, Yansheng Li, Xiaobing Liu, Guanghua Wang, Caixian Tang, Zhenhua Yu, Xiaojuan Wang, Stephen J. Herbert

**Affiliations:** 1 Key Laboratory of Mollisols Agroecology, Northeast Institute of Geography and Agroecology, Chinese Academy of Sciences, Harbin, China; 2 Centre for AgriBioscience, La Trobe University, Melbourne Campus, Bundoora, Vic, Australia; 3 Stockbridge School of Agriculture, University of Massachusetts, Amherst, MA, United States of America; Huazhong Agriculture University, CHINA

## Abstract

Understanding how elevated CO_2_ affects dynamics of nodal leaf growth and N assimilation is crucial for the construction of high-yielding canopy via breeding and N management to cope with the future climate change. Two soybean cultivars were grown in two Mollisols differing in soil organic carbon (SOC), and exposed to ambient CO_2_ (380 ppm) or elevated CO_2_ (580 ppm) throughout the growth stages. Elevated CO_2_ induced 4–5 more nodes, and nearly doubled the number of branches. Leaf area duration at the upper nodes from R5 to R6 was 4.3-fold greater and that on branches 2.4-fold higher under elevated CO_2_ than ambient CO_2_, irrespective of cultivar and soil type. As a result, elevated CO_2_ markedly increased the number of pods and seeds at these corresponding positions. The yield response to elevated CO_2_ varied between the cultivars but not soils. The cultivar-specific response was likely attributed to N content per unit leaf area, the capacity of C sink in seeds and N assimilation. Elevated CO_2_ did not change protein concentration in seeds of either cultivar. These results indicate that elevated CO_2_ increases leaf area towards the upper nodes and branches which in turn contributes yield increase.

## Introduction

Given that the rate of increase of atmospheric CO_2_ concentration has accelerated during the past two centuries [1,2], and the CO_2_ concentration is expected to climb up to 800 μL L^-1^ by the end of this century [3,4], crop yield in the agricultural system is likely to increase [5–8]. For example, positive responses of soybean yield to elevated CO_2_ (eCO_2_) have been recorded in many studies [9–11], but the extent of increase varied among the cultivars. In a glasshouse experiment, eCO_2_ increased soybean yields by 20 to 90% when nine cultivars were compared [12]. Similarly, the magnitude of yield increase in response to eCO_2_ was up to 24% when 18 genotypes of soybean were grown in a FACE (Free Air CO_2_ Enrichment) facility [11].

Several agronomic and physiological characteristics likely contribute to the stimulation of soybean yield under eCO_2_. One of these is the increased leaf area under eCO_2_. This is because the response of leaf growth to eCO_2_ changes canopy structure and the capability of C assimilation of the entire canopy [12], contributing to the increase in biomass production and subsequent grain yields. Moreover, increasing N uptake in response to eCO_2_ is critical to the yield increase, since eCO_2_ improves N acquisition from soil and N_2_ fixation of soybean, providing more N for photosynthesis and biomass production [13–15].

Unlike other crops such as rice and wheat, vegetative and reproductive stages of soybean overlap each other, creating and sustaining C sink of the seed along the plant axis [16]. Thus, how the temporal and spatial distribution of leaf area in response to eCO_2_ is likely to determine grain yield along the main axis and branches. Moreover, these responses would also be associated with N assimilation because N is the major component of photosynthesis-related enzymes, such as ribulose biphosphate carboxylase/oxygenase (RUBISO) [17, 18]. Although the distribution of nodal leaf area in soybean plants has been specifically studied under different farming and environmental conditions [19, 20], little information is available on the development patterns of nodal leaf area in eCO_2_ environments.

As a major crop, soybean is widely grown in the region of Mollisols in northeast China, the world’s third largest contiguous body of Mollisols [21]. The content of soil organic carbon (SOC) differs widely among different regions of Mollisols, which may affect the capability of a soil to supply N and hence influence crop response to eCO_2_. A study by McGrah and Lobell [22] showed that the regional yield response to increased CO_2_ varied due to environmental conditions. However, it has not been experimentally tested whether SOC affects the response of soybean yield to eCO_2_ in Mollisols.

The objective of this study was to investigate the effect of eCO_2_ on yield-related characteristics including dynamics of nodal leaf area and N uptake in two soybean cultivars grown in Mollisols differing in SOC. We hypothesized that eCO_2_ would greatly enhance yield with changes in leaf distribution along the axis and plant N assimilation, but the extent may depend on cultivar and soil type due to atmosphere-plant-soil interactions.

## Materials and methods

### Experimental design and plant growth

The experiment consisted of two levels of CO_2_, two soybean cultivars and two Mollisols in a split-plot design with CO_2_ as the main plot, and soybean cultivar and soil as sub-plot treatments. Each treatment had four replicates. The CO_2_ treatment was achieved using open top chambers (OTC) (8 m^2^ each) located at the Northeast Institute of Geography and Agroecology (45°42´N, 126°38´E), Chinese Academy of Sciences, Harbin, China. There were four OTCs for eCO_2_ (580 ppm) and four for ambient CO_2_ (380 ppm). A digital CO_2_-regulating system (Beijing VK2010, China) was installed to monitor the CO_2_ level in each eCO_2_ OTC and automatically regulate the supply of CO_2_ gas (99.9%) to achieve CO_2_ concentrations at 580±30 ppm for eCO_2_. Elevated CO_2_ was supplied for 24 h day^-1^. The soybean (*Glycine max* L. Merr.) cultivars were Suinong 14 (Maturity Group 0) and Dongsheng 7 (Maturity Group 0), which have been widely grown in Mollisols regions of Northeast China, but differs in the year of release with Suinong 14 in 1996 and Dongsheng 7 in 2012 [23, 24].

Two soils (Mollisol) differing in SOC were collected at a depth of approximately 0 to 10 cm from two farmlands which were located at Lishu county, Liaoning Province (43°20´N, 124°30´E) and Wuchang county, Heilongjiang Province (45°21´N, 126°39´E). The soil from Lishu had SOC of 10.4 mg g^-1^, total N of 0.8 g kg^-1^ and available N of 75 mg kg^-1^, while the soil from Wuchang had SOC of 45.5 mg g^-1^, total N of 3.4 g kg^-1^ and available N of 167 mg kg^-1^. The soils were air-dried and sieved through a 4-mm sieve. Eighteen kg soil was filled into each of 19-L pots, and compacted to a bulk density of 1.1 g cm^-3^. Basal nutrients were applied at the following rates (mg kg^-1^): urea, 217; KH_2_PO_4_, 219; CaCl_2_.2H_2_O, 167; MgSO_4_.7H_2_O, 43; Fe-EDTA, 9; ZnSO_4_ 6; H_3_BO_3_, 0.7; MnSO_4_.H_2_O, 10; CuSO_4_.5H_2_O, 2; CoSO_4_.7H_2_O, 0.3; and Na_2_MoO_4_.2H_2_O, 0.2. The nutrients were thoroughly mixed with the soil.

**Fig 1:**
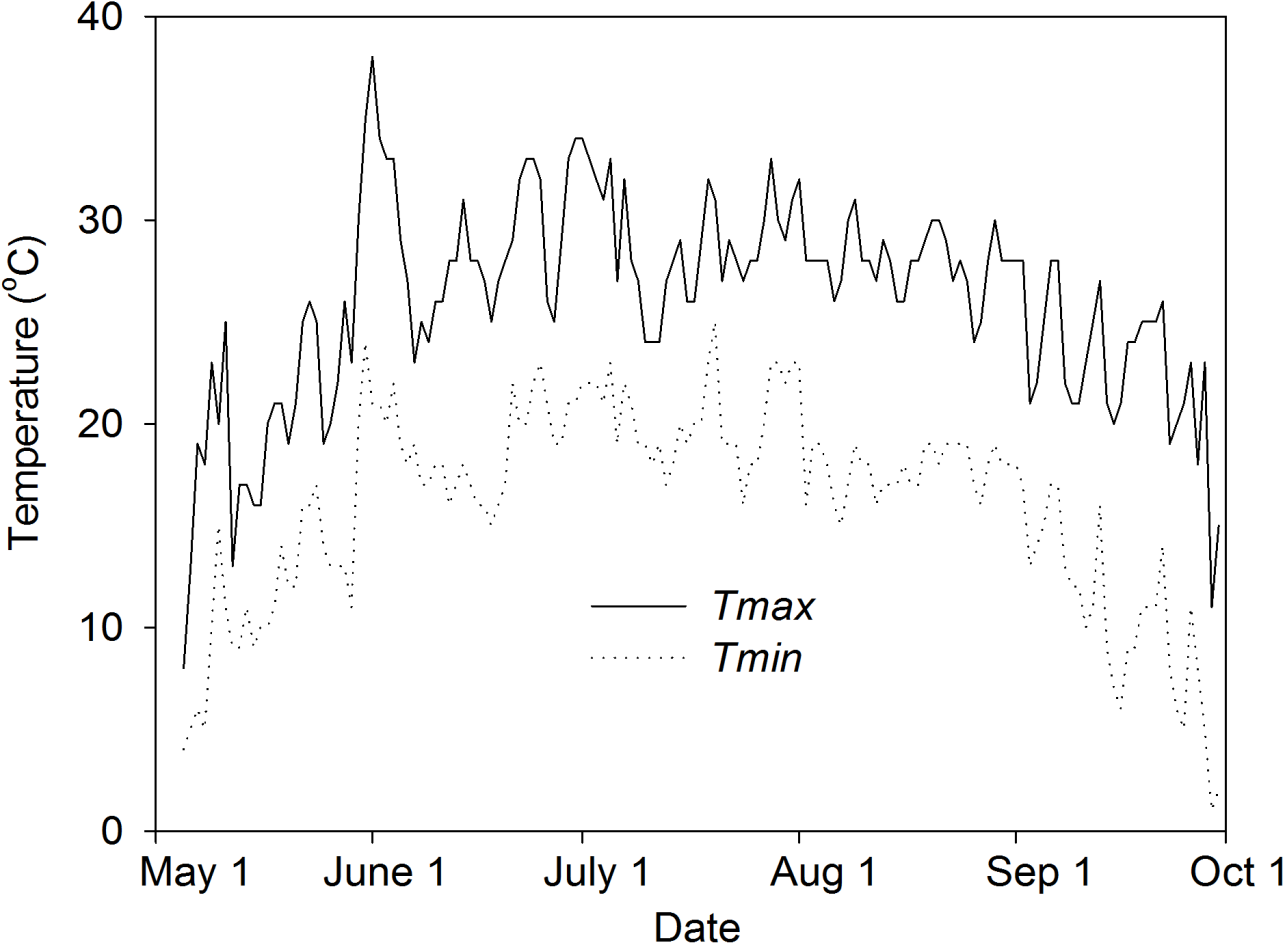
Daily minimal (*Tmin*) and maximal (*Tmax*) temperatures inside the Open Top Chamber (OTC) (average of eight OTCs) during the experimental period from 5^th^ of May to 1^st^ of October, 2014.

Nine seeds of uniform size were sown into each pot on May 5, 2014. The plants were thinned to three per pot 10 days after emergence. Each OTC had one investigated pot in the middle of eight other pots to establish a canopy density of 33 plants m^-2^, as indicated by Oikawa *et al*. [25]. The average OTC-inside temperatures across eight OTCs during the experimental period are shown in Fig 1. The variation of temperature between OTCs was less than 0.5°C and there was no difference in temperature in OTCs between aCO_2_ and eCO_2_. Soil water content was maintained at 80±5% of field capacity by weighing.

### Measurements

At the stages of R4 (full pod setting) (Day 68), R5 (initial pod filling) (Day 85), and R6 (full seed) (Day 101), leaf area at each node position on the main axis and each branch corresponding to the main axis node was measured using a portable laser leaf area analyzer (CI-203, CID Biosciences Inc., Camas USA). Chlorophyll content was determined on antepenultimate fully expanded leaves using a chlorophyll meter (CCM-300, Opti-Sciences Inc., Hudson, USA). Two leaflets that were fully expanded at the penultimate node were then sampled from two plants in the centre of the investigated pot, oven dried and stored for later measurements.

At the R8 stage (full maturity), shoots were removed at the soil surface and shoot biomass was recorded. Detailed data of yield components including pod number, seed number and seed dry weight were recorded according to node position and corresponding branches. Shoots were then separated into stems plus pod walls, and seeds. All plant samples were dried at 70°C for 72 h and then finely ground (≤0.2 mm). The concentration of N in plant tissues was determined using an Elementar CNS analyser (Vario EL III, Elementar Analysensysteme GmbH, Germany).

### Calculations

Leaf area duration (LAD) gives an indication of solely days of green area [26] and was calculated using the following equation.

$$\mathrm{LAD}=\left(\mathrm{Leaf}\;\mathrm{are}a_{1}+\mathrm{Leaf}\;\mathrm{are}a_{2}\right)\times \left(T_{2}\text{-}T_{1}\right)/2$$

Where Leaf area_1_ and Leaf area_2_ are the leaf area at the growth stages T_1_ and T_2_, respectively [27].

Because of leaf senescence at maturity, harvest index (HI) was calculated as the weight ratio of seed to stem plus pod biomass at maturity [12]. Tissue N content was calculated by multiplying the N concentration in the tissue by tissue weight. Seed protein content was estimated as the N concentration in seed multiplying by an N conversion factor of 6.25 [28]. Nitrogen harvest index (NHI) was defined as N content in seed relative to total above-ground N at maturity [29]. Nitrogen content per unit leaf area was the ratio of N content of leaf to the leaf area [30, 31].

### Statistical analysis

Statistical analyses were performed on variables using Genstat 13 (VSN International, Hemel Hemspstead, UK). Analysis of variance (ANOVA) was used to determine the effects of CO_2_, cultivar and soil, and their interactions on grain yield, yield components, shoot biomass, HI, total node number, branch number, shoot N concentration and content, and NHI. Protected ANOVA tests of LSD were used to assess the differences between treatment means [32]. Since there was no significant interaction between soil and CO_2_ in terms of grain yield and leaf area (*P* > 0.05), the data sets from two soils were combined for the distributions of seed weight and leaf area along the main axis.

Pearson correlations between grain yield and measured parameters and between N content per unit leaf area and chlorophyll content were performed. The significance for these correlations was evaluated by student’s *t* test at the 0.05 probability level.

## Results

### Seed yield and yield components

Elevated CO_2_ increased seed yields of Dongsheng 7 and Suinong 14 by 35% and 13%, respectively, when they were grown in the low-SOC Mollisol (Table 1). A similar trend was found in the high-SOC Mollisol with 40% and 28% of increases under eCO_2_ for Dongsheng 7 and Suinong 14, respectively. The interaction between CO_2_ and cultivar was significant (*P* < 0.05) (Table 1).

**Table 1:** The effect of elevated CO_2_ on grain yield, pod number, seed number, seed size, node number and branch number of soybean. Two soybean cultivars, Suinong 14 and Dongsheng 7, were grown in Mollisols. Plants were exposed to ambient (aCO_2_) (380 ppm) or elevated CO_2_ (eCO_2_) (580 ppm). Values are means ± standard error of variables across the four replicates, and the statistical significance levels for the effects of CO_2_, cultivar, soil and their interaction are shown. SOC, soil organic C.

Mollisols	Cultivar	Grain Yield (g plant^-1^)		Pod Number (No. plant^-1^)		Seed Number (No. plant^-1^)		Seed Size (mg g^-1^)		Node Number (No. plant^-1^)		Branch Number (No. plant^-1^)	
		aCO_2_	eCO_2_	aCO_2_	eCO_2_	aCO_2_	eCO_2_	aCO_2_	eCO_2_	aCO_2_	eCO_2_	aCO_2_	eCO_2_
Low SOC	Suinong 14	23.1±0.56	26.1±0.77	33.4±2.62	57.1±0.48	83.0±6.43	116.0±2.22	284±14.0	225±7.8	16.7±0.80	21.3±0.50	2.0±0.58	4.0±0.58
	Dongsheng 7	18.7±0.79	25.3±0.98	32.9±0.11	56.3±1.07	77.8±1.64	115.3±0.31	241±12.0	219±9.1	15.3±0.39	19.0±0.37	1.3±0.33	3.0±1.00
High SOC	Suinong 14	20.1±0.36	25.6±1.35	34.1±1.37	54.2±1.44	83.1±7.32	110.0±6.70	257±8.9	233±3.1	16.3±0.33	20.3±0.40	2.3±0.33	4.0±1.00
	Dongsheng 7	18.5±0.32	26.0±0.29	31.6±1.83	48.7±0.77	77.0±0.69	110.0±3.98	241±3.5	237±10.1	15.3±0.49	19.3±0.67	2.0±0.58	4.0±0.58
LSD (*P* = 0.05)		2.1		4.3		8.7		28		1.1		2.0	
*P* values													
CO_2_		<0.001		< .001		<0.001		<0.001		0.001		<0.001	
Cultivar		0.016		0.104		0.301		0.031		0.305		0.002	
Soil		0.095		0.062		0.871		0.968		0.305		0.990	
CO_2_ × Cultivar		0.009		0.252		0.436		0.045		0.990		0.536	
CO_2_ × Soil		0.262		0.012		0.081		0.067		0.990		0.536	
Cultivar × Soil		0.034		0.067		0.213		0.185		0.490		0.224	
CO_2_ × Cultivar × Soil		0.773		0.297		0.477		0.535		0.728		0.076	

Elevated CO_2_ increased the number of pods and seeds by averages of 63% and 41%, respectively (Table 1). The main effects of soil and cultivar were not significant. However, eCO_2_ decreased seed size. For example, in the low-SOC Mollisol, eCO_2_ decreased the seed size by 21% for Suinong 14 and 7% for Dongsheng 7, leading to a significant interaction between CO_2_ and cultivar (Table 1).

Elevated CO_2_ increased node number from 16 to 21 for Suinong 14, and from 15 to 19 for Dongsheng 7. The main effect of soil and the interaction between CO_2_ and cultivar on node number were not significant. Elevated CO_2_ almost doubled the number of branches in both cultivars (Table 1).

**Fig 2:**
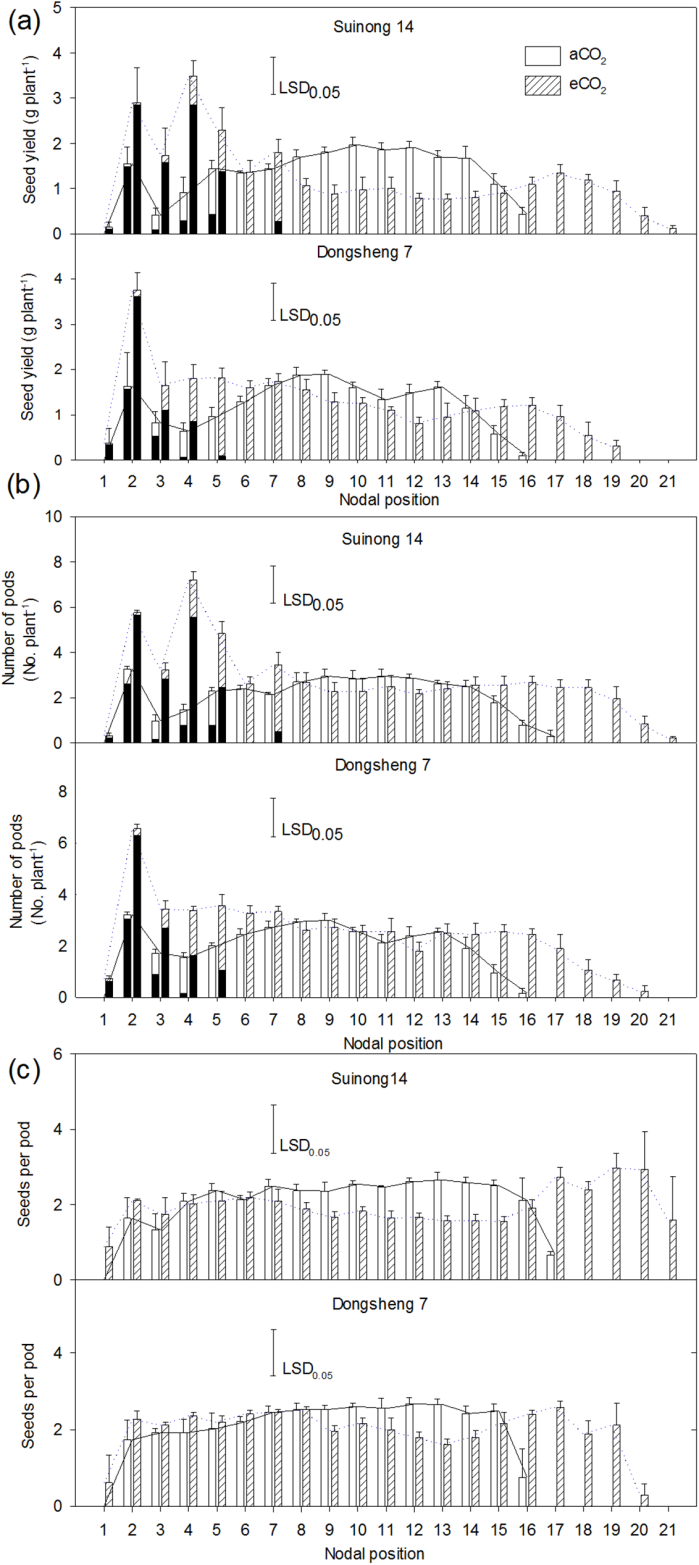
Distribution of seed weight (a), number of pods (b) and number of seeds per pod (c) along the main axis (The values on branches were highlighted with black colour in bars corresponding to the main axis node) of soybean cultivars, Suinong 14 and Dongsheng 7, grown in Mollisols under ambient (aCO_2_) (380 ppm) and elevated CO_2_ (eCO_2_) (580 ppm). The number of nodal position counts from the bottom (1) to the top (21) of the main axis. Values are means ± standard error of variables across the twelve replicates. The horizontal LSD bars (*P* = 0.05) in each panel are also shown.

Elevated CO_2_ dramatically altered the distribution of seed weight along the plant axis. Elevated CO_2_ resulted in greater seed weight distributed towards the newly-formed nodes and branches but less seed distributed between the 8^th^ and 14^th^ nodes compared to aCO_2_ (Fig 2A). In proportion, 14–17% of yield of Suinong 14 and 6–9% of yield of Dongsheng 7 were allocated to the new nodes from the 17^th^ to 21^st^ node under eCO_2_. The yield on the branches of Suinong 14 increased from 6% under aCO_2_ to 34% under eCO_2_, and this value increased from 9% to 25% in Dongsheng 7. The distribution of pod number on the main axis had a similar trend to the seed weight (Fig 2B). Elevated CO_2_ did not affect seed number per pod (Fig 2C).

Elevated CO_2_ increased plant biomass at maturity by 25% for Suinong 14, and by 36% for Dongsheng 7 (Table 2). The eCO_2_ did not affect the harvest index for Dongsheng 7 but decreased it for Suinong 14 (Table 2).

**Table 2:** The effect of elevated CO_2_ on shoot biomass, harvest index (HI), shoot N concentration, shoot N content, N harvest index (NHI) of soybean. Two soybean cultivars, Suinong 14 and Dongsheng 7, were grown Mollisols. Plants were exposed to ambient (aCO_2_) (380 ppm) or elevated CO_2_ (eCO_2_) (580 ppm). Values are means ± standard error of variables across the four replicates, and the statistical significance level P values for the effects of CO_2_, cultivar, soil and their interaction are shown. SOC, soil organic C.

Mollisols	Cultivar	Shoot Biomass (g plant^-1^)		HI		Shoot N concentration (mg g^-1^)		Shoot N Content (mg plant^-1^)		Grain Protein (mg g^-1^)		NHI	
		aCO_2_	eCO_2_	aCO_2_	eCO_2_	aCO_2_	eCO_2_	aCO_2_	eCO_2_	aCO_2_	eCO_2_	aCO_2_	eCO_2_
Low SOC	Suinong 14	43.3±0.64	54.2±1.43	0.53±0.01	0.48±0.01	43.5±1.07	36.9±0.77	1882±58	2001±86	391±2.4	384±10.5	0.77±0.01	0.80±0.02
	Dongsheng 7	37.6±1.12	51.3±1.37	0.49±0.01	0.49±0.01	42.4±1.60	37.3±0.40	1594±87	1915±55	392±5.1	384±5.0	0.74±0.02	0.81±0.01
High SOC	Suinong 14	39.1±0.58	49.4±1.35	0.51±0.01	0.49±0.01	40.5±0.96	39.4±2.50	1584±49	1945±107	391±9.3	389±1.9	0.79±0.01	0.82±0.01
	Dongsheng 7	37.0±0.52	50.0±0.39	0.50±0.01	0.52±0.01	43.2±1.28	41.2±0.65	1597±25	2063±19	378±5.2	383±5.0	0.70±0.02	0.78±0.01
LSD (*P* = 0.05)		1.4		0.01		3.9		202		19		0.04	
*P* values													
CO_2_		<0.001		0.045		0.001		<0.001		0.747		<0.001	
Cultivar		0.003		0.829		0.320		0.220		0.496		0.002	
Soil		<0.001		0.586		0.248		0.304		0.743		0.523	
CO_2_ × Cultivar		0.034		<0.001		0.286		0.857		0.636		0.047	
CO_2_ × Soil		0.343		0.045		0.138		0.035		0.225		0.974	
Cultivar × Soil		0.011		0.052		0.003		0.182		0.368		0.019	
CO_2_ × Cultivar × Soil		0.826		0.920		0.378		0.525		0.452		0.760	

### Leaf area distribution

**Fig 3:**
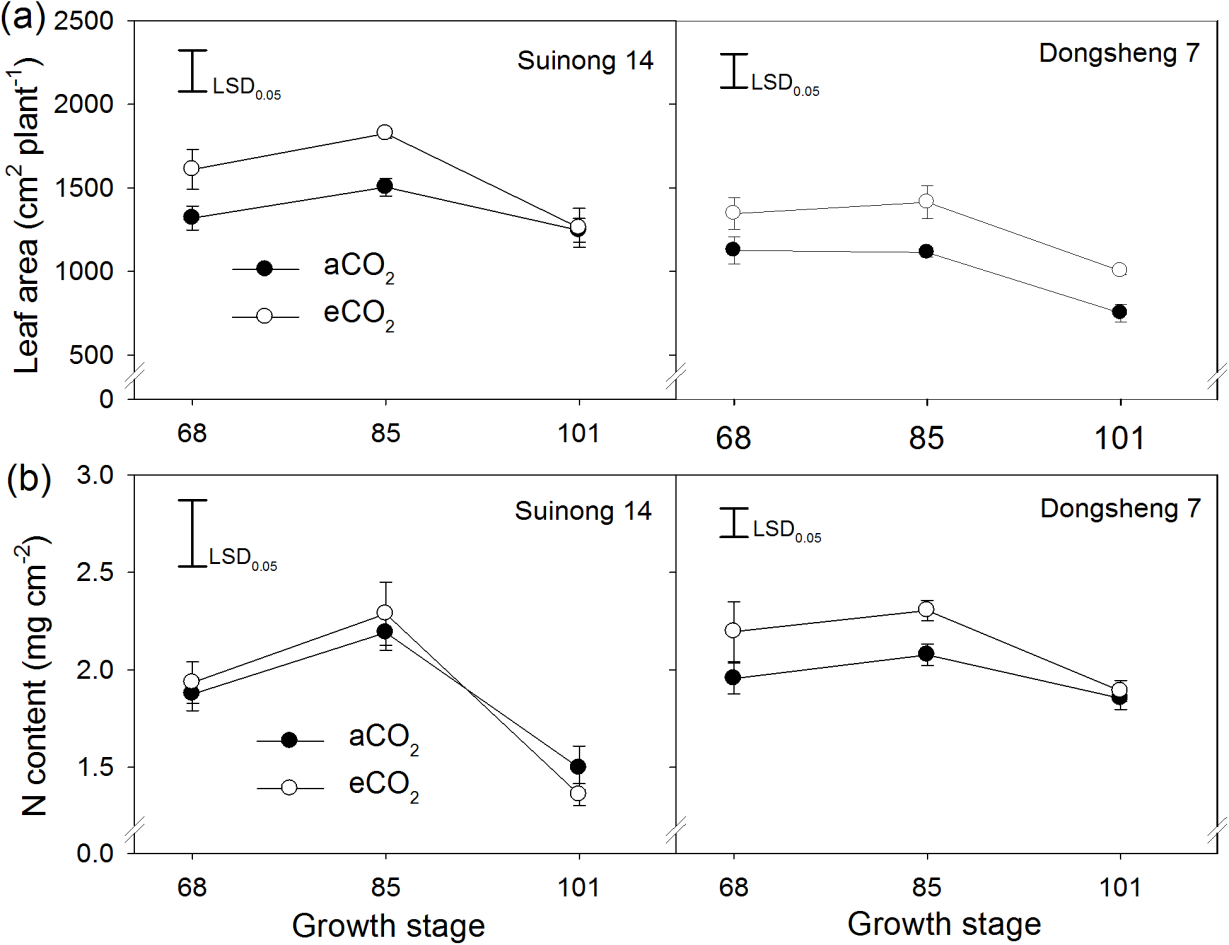
The effect of elevated CO_2_ on leaf area (a) and N content per unit leaf area (b) during the period from the R4 (68 d), R5 (85 d) to R6 (101 d) stages. Two soybean cultivars, Suinong 14 and Dongsheng 7, were grown in Mollisols under ambient (aCO_2_) (380 ppm) and elevated CO_2_ (eCO_2_) (580 ppm). Values are means ± standard error of variables across the four replicates. The vertical LSD bars (*P* = 0.05) in each panel are shown.

Elevated CO_2_ resulted in 9–37% increase in leaf area during the period from R4 to R5 (Fig 3A). By R6, however, leaf area of Suinong 14 did not differ between aCO_2_ and eCO_2_, while that of Dongsheng 7 was still greater under eCO_2_. The response of leaf area to eCO_2_ was similar between the two soils.

**Fig 4:**
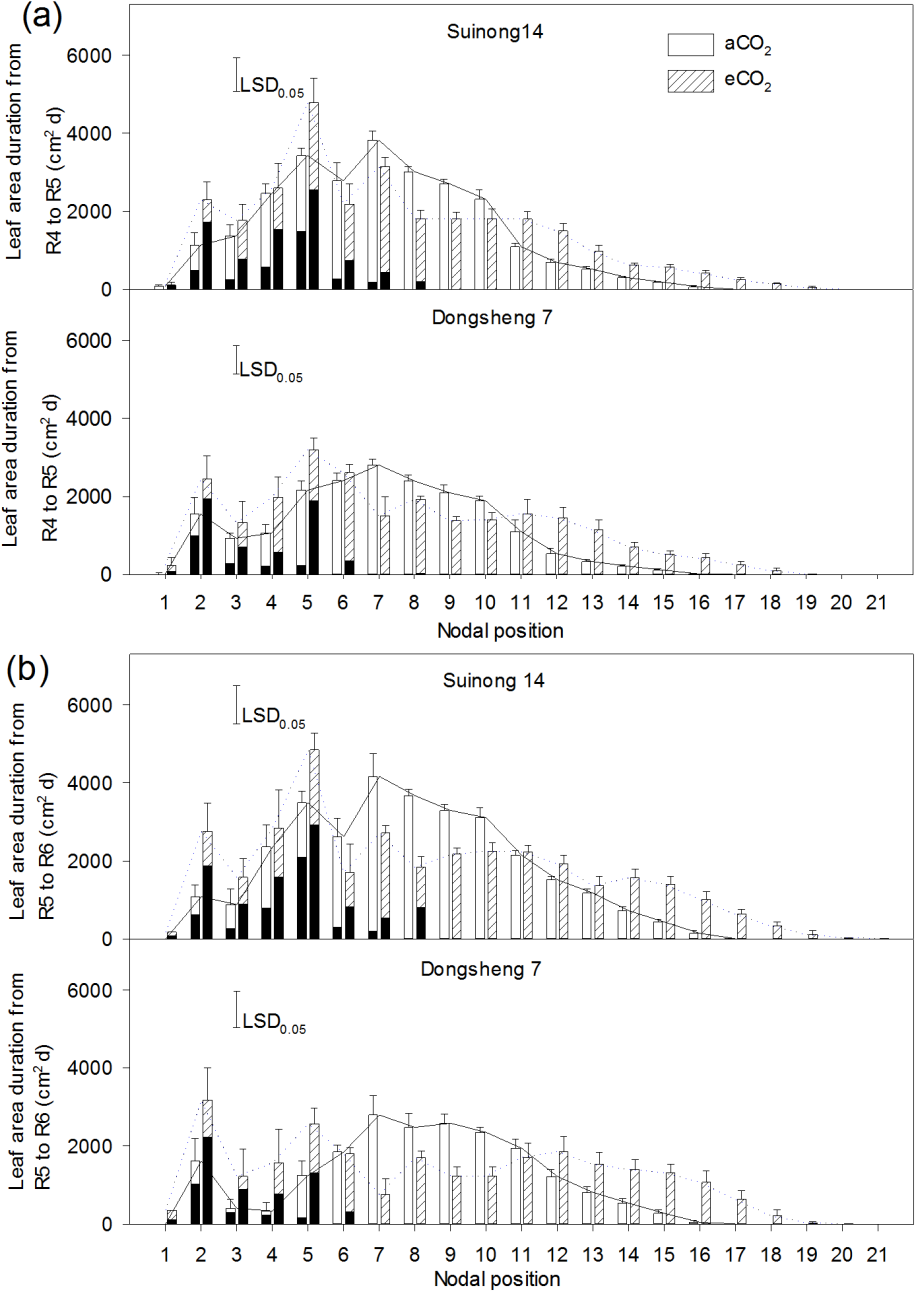
Distribution of leaf area duration from R4 to R5 (a) and from R5 to R6 (b) along the main axis (values on branches corresponding to the main axis node were shown as black bars) of soybean cultivars, Suinong 14 and Dongsheng 7, grown in Mollisols under ambient (aCO_2_) (380 ppm) and elevated CO_2_ (eCO_2_) (580 ppm). The number of nodal position counts from the bottom (1) to the top (21) of the main axis. Values are means ± standard error of variables across the twelve replicates. The vertical LSD bars (*P* = 0.05) in each panel are shown.

Elevated CO_2_ greatly increased leaf area duration at upper nodes and on branches, and this effect was more pronounced in the R5 to R6 period than that in the R4 to R5 period (Fig 4A and 4B). From R5 to R6, leaf area duration on the upper nodes, especially from the 15^th^ to 19^th^ node, was 4.3-fold greater, and that on branches 2.4-fold higher under eCO_2_ than aCO_2_, irrespective of cultivar and soil type. In contrast, eCO_2_ reduced leaf area duration at the middle nodes, in particular, from 6^th^ to 10^th^ nodes. The leaf area duration of Suinong 14 was higher than that of Donsheng 7 (Fig 4B).

### Leaf N and leaf chlorophyll content

**Fig 5:**
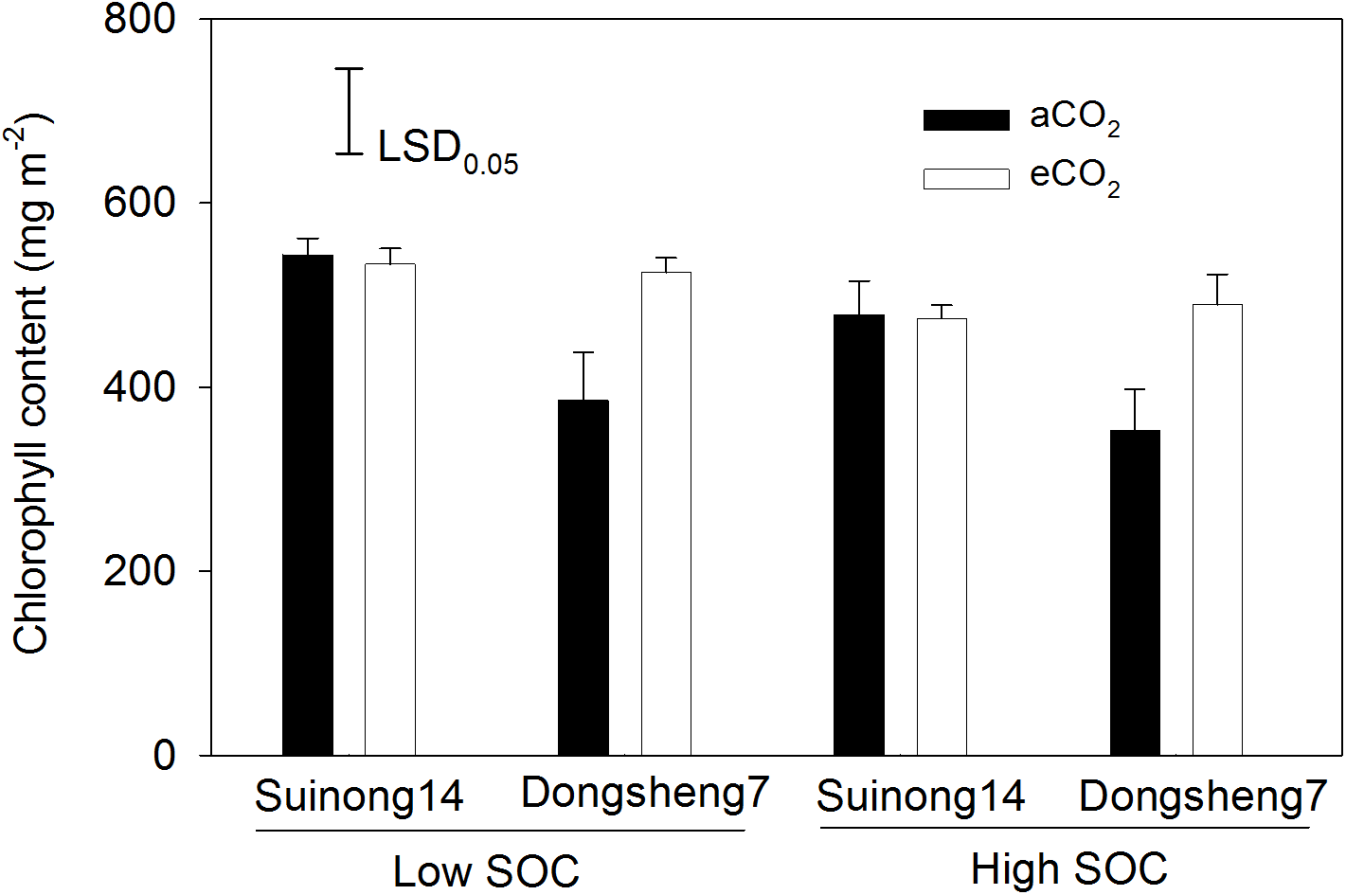
The effect of elevated CO_2_ on chlorophyll content of antepenultimate fully expanded leaf at R5. Two soybean cultivars, Suinong 14 and Dongsheng 7, were grown in low- and high-SOC Mollisols under ambient (aCO_2_) (380 ppm) and elevated CO_2_ (eCO_2_) (580 ppm). Values are means ± standard error of variables across the twelve replicates. The vertical LSD bars (*P* = 0.05) are shown. SOC, soil organic C.

**Fig 6:**
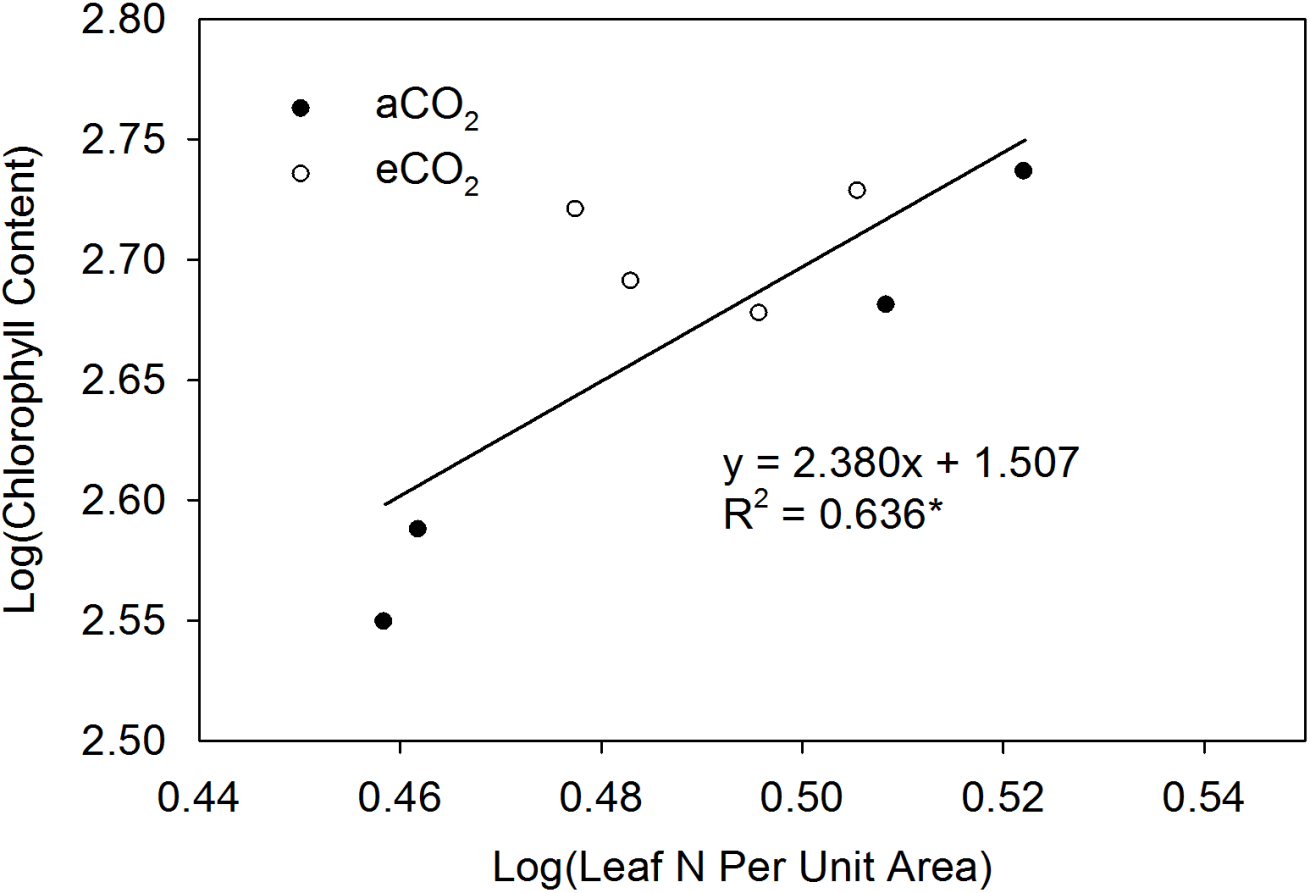
Relationship between N content per unit leaf area and leaf chlorophyll content at R5. * indicates *P* < 0.05.

The effects of eCO_2_ on N content per unit leaf area and leaf chlorophyll content varied between the two cultivars. Elevated CO_2_ did not affect N content per unit leaf area in Suinong 14 across growth stages from R4 to R6, but increased it in Dongsheng 7, especially at R4 and R5 (Fig 3B). Soil did not affect leaf N content per unit leaf area or chlorophyll content. While there was no difference in chlorophyll content in leaves of Suinong 14 between aCO_2_ and eCO_2_, eCO_2_ increased the leaf chlorophyll content of Dongsheng 7 by an average of 37% (Fig 5). Irrespective of CO_2_ treatment, N content per unit leaf area correlated positively with leaf chlorophyll content (*P* < 0.05) (Fig 6).

### N uptake, N harvest index and protein concentration

Shoot N concentration was on average 15% lower under eCO_2_ than under aCO_2_ (Table 2). Neither cultivar nor soil had a significant effect on the N concentration. Elevated CO_2_ increased shoot N uptake, and this positive response to eCO_2_ varied between the cultivars, resulting in a significant interaction between CO_2_ and cultivar (Table 2). In the low-SOC Mollisols, eCO_2_ increased the N uptake by 6% in Suinong 14 and 20% in Dongsheng 7. In the high-SOC Mollisol, the response of shoot N content to eCO_2_ was also greater in Suinong 14 than in Dongsheng 7.

Protein concentration was not affected by eCO_2_ in either Suinong 14 or Dongsheng 7 with an average of 387 mg g^-1^ (Table 2). Elevated CO_2_ did not influence the N harvest index in Suinong 14, but increased it in Dongsheng 7 by 10%. The trend was similar in the two soils.

## Discussion

The significant response of grain yield to eCO_2_ was largely due to the increase of seed number rather than the seed size. In this study, eCO_2_ increased the numbers of seeds and pods but decreased or did not affect seed size (Table 1). Similarly, in a FACE experiment, Bishop *et al*. [11] showed no significant effect of CO_2_ on 100-seed weight, but a 9% increase of grain yield, indicating that seed number greatly contributed to the yield gain in response to eCO_2_. Furthermore, it has been reported that increased pod weight was associated with increased grain yield, when soybean plants were exposed to 710 ppm of CO_2_ [12].

The investigation on where the increase of yield occurs in the canopy of soybean is fundamental to understanding the mechanisms of yield response to eCO_2_. Elevated CO_2_ altered the spatial distribution of yield components and subsequent yield (Fig 2). Compared to aCO_2_, the increased number of nodes and branches (Table 1), and the greater weight (Fig 2) and proportion of seeds (data not shown) at the corresponding parts under eCO_2_ indicate that the newly-formed nodes and branches were the main sinks for the extra photosynthates under eCO_2_. This point is also supported by a number of previous studies. Ziska *et al*. [12] found that the increased axillary branching under eCO_2_ was associated with yield sensitivity to eCO_2_. The observation here is in accordance with the findings in rice and wheat of which tillering was correlated with the ability of grain yield to respond strongly to eCO_2_ [33, 34]. Moreover, in a SoyFACE experiment, the increase in node number was observed in soybean grown in a Typic Endoaquoll [16, 35]. Thus, eCO_2_ enhances the ability of soybean to produce additional seeds on extended axis and axillary branches. The contribution of seeds on branches to the yield increase was higher than that at increased nodes (Fig 2).

The periodic pattern of nodal leaf growth has been shown to greatly affect available C for its input into reproductive sinks [36]. The greater leaf area of the whole plant during the period from R4 to R6 under eCO_2_ than aCO_2_ (Fig 3) would increase the number of flowers and pods via increased supply of photosynthates [37, 38]. Spatially, the greater leaf area duration at the upper nodes and on branches under eCO_2_ than under aCO_2_ (Fig 4) contributed to the photosynthetic stimulation under eCO_2_ and corresponded with the distributions of pods and seed weight (Fig 2). In particular, the effect of CO_2_ on leaf area duration was greater from R5 to R6 than from R4 to R5, indicating that nodal leaf area during late reproductive stages determined the yield gain under eCO_2_. This is evident of a significant relationship between nodal leaf area duration from R5 to R6 and respective yield observed (Data not shown). Rascher *et al*. [39] observed that eCO_2_ stimulated leaf-level electron transport in light reactions of photosynthesis in the soybean canopy and increased biomass production, supporting that the eCO_2_-mediated change in canopy structure favors yield formation at the physiological level. While eCO_2_ increased leaf area duration at the upper nodes, it decreased leaf area duration (Fig 4) and proportion at the middle nodes (data not shown). This suggests that the C budget for leaf expansion under eCO_2_ is likely limited, and is mainly used for development of new leaves and seeds at other nodes.

Sufficient N uptake and transport to the seed are especially important for the yield gain under eCO_2_, given that N demand is intrinsically great in soybean, and adequate supply of N is consequently vital to attain yield potential [17, 18]. Greater shoot N content and N harvest index under eCO_2_ (Table 2), and their linear correlations with grain yield (data not shown) indicate the importance of N in the yield response to eCO_2_. In addition, the reduction in shoot N concentration under eCO_2_ suggests a dilution effect as well as increased N demands by the plant.

Besides grain yield, grain quality is expected to be affected by eCO_2_. A meta-analysis based on 228 experimental observations on different crops indicates a reduction on protein concentrations when grown at eCO_2_ (540–958 ppm) compared with ambient CO_2_ (315–400 ppm) [40]. Hao *et al*. [41] found that eCO_2_ decreased protein concentration of soybean grain by 3.3% in a FACE experiment. In the present study, eCO_2_ did not significantly affect the protein (or N) concentration in soybean grains (Table 2), indicating that N supply was adequate to maintain the grain quality. Myers *et al*. [42] also indicated that there was no significant CO_2_ effect on soybean protein concentration. The lower N concentration in shoot and higher N harvest index under eCO_2_ (Table 2) indicate that eCO_2_ facilitated the translocation of N into grains. Furthermore, eCO_2_ might enhance N assimilation during the reproductive stages [43], contributing to the high N harvest index.

The two cultivars differed in the yield response to eCO_2_, with yield increase being greater in Dongsheng 7 than Suinong 14. A similar result was found in soybean that 18 genotypes varied in the responses of their grain yields to eCO_2_ from nil to a 20% increase [11]. The cultivar variation in yield response to eCO_2_ in this study might be attributed to several reasons.

The first would be the enlarged sink of photosynthates in Dongsheng 7, since this cultivar had greater responses to eCO_2_ in pod number and harvest index (Tables 1 and 2). The additional photosynthates produced under eCO_2_ can be allocated to this sink organ to form more seeds [44, 45]. Bishop *et al*. [11] observed that there was a positive correlation between changes in partitioning coefficient of photosynthates and yield under eCO_2_. Moreover, eCO_2_ did not significantly change seed size of Dongsheng 7 but decreased that of Suinong 14 (Table 1). This indicates that the translocation of photosynthates to the seed were not sufficient in Suinong 14, limiting the response of this cultivar to eCO_2_.

Secondly, the increase of N content per unit leaf area in Dongsheng 7 under eCO_2_ is likely associated with greater yield gain compared to Suinong 14 (Fig 3). This is because the dominant contributor of the leaf photosynthetic function is leaf N status which in turn affects chlorophyll synthesis [46, 47]. With 46 soybean cultivars, Jin *et al*. [31] found a significant positive relationship between N content per unit leaf area and biomass accumulation. This present study showed a significant relation of N content per unit leaf area with chlorophyll concentration (Fig 6). Moreover, the chlorophyll content of Dongsheng 7 had a greater increase in response to eCO_2_ compared to aCO_2_ (Fig 5). Thus, under eCO_2_, greater increase of N content per unit leaf area in Dongsheng 7 resulted in greater responses in biomass accumulation and grain yield.

Thirdly, the more sensitive response to eCO_2_ of gas exchange through leaf stomata would lead to greater yield gain in Dongsheng 7. As many modern soybean cultivars with high stomatal conductance have been bred to gain high yields, newly released Dongsheng cultivars had higher stomatal conductance than Suinong cultivars [48, 49], and favored CO_2_ uptake and photosynthesis under eCO_2_. Thus, the CO_2_ effect would be greater in Dongsheng 7 than Suinong 14. However, the cultivar with the greater gas exchange capacity is more sensitive to ozone that deteriorate photosynthetic metabolism and crop yield [50], especially in soybean cultivars with high stomatal conductance [48, 51]. Nevertheless, the average concentration of atmospheric ozone at the experimental region was only 19.1 ppb, below the threshold of 40 ppb where impacts on primary metabolism are frequently observed [48]. The ozone and CO_2_ interaction on the cultivar yield response would be minimal in this study.

Finally, the responses of N uptake and N harvest index to eCO_2_ would contribute to the yield increase in Dongsheng 7 (Table 2). Greater N uptake and N harvest index are required to satisfy the N demand in seed development under eCO_2_, favoring yield formation [9, 17, 18]. Based on an analysis of 108 studies, soybean exhibits a linear relationship between grain yield and total N uptake in the above-ground biomass [18]. Thus, the genotypic variation in N-uptake efficiency and N-use efficiency for yield formation under eCO_2_ requires further investigation.

Soil type did not affect the responses of biomass production, N uptake, leaf area and final grain yield to eCO_2_, indicating that the variation in the properties of Mollisols in different regions might not be a major factor influencing the production of soybean in a high CO_2_ environment. This is probably because the nutrient supply was adequate for plant growth, especially in the N_2_-fixing soybean. The symbiotic process might offset the impact of soil type on plant response to eCO_2_ in this study. However, Sakurai *et al*. [10] reported that the effect of high CO_2_ concentration on soybean yield varied between different regions, with 4.3%, 7.6% and 5.1% of increase in the USA, Brazil and China, respectively, due to the increase of atmospheric CO_2_ concentration. This variation may be attributed to the difference in soil type and climate, and soybean genotypes. Further research is needed to quantify the contribution of photosynthetic C to N assimilation, especially N_2_ fixation under eCO_2_, and the cultivar variation in this symbiotic association in response to eCO_2_. This would be essential for the N management that creates a high yielding canopy for soybean in the future.

## Conclusions

**Fig 7:**
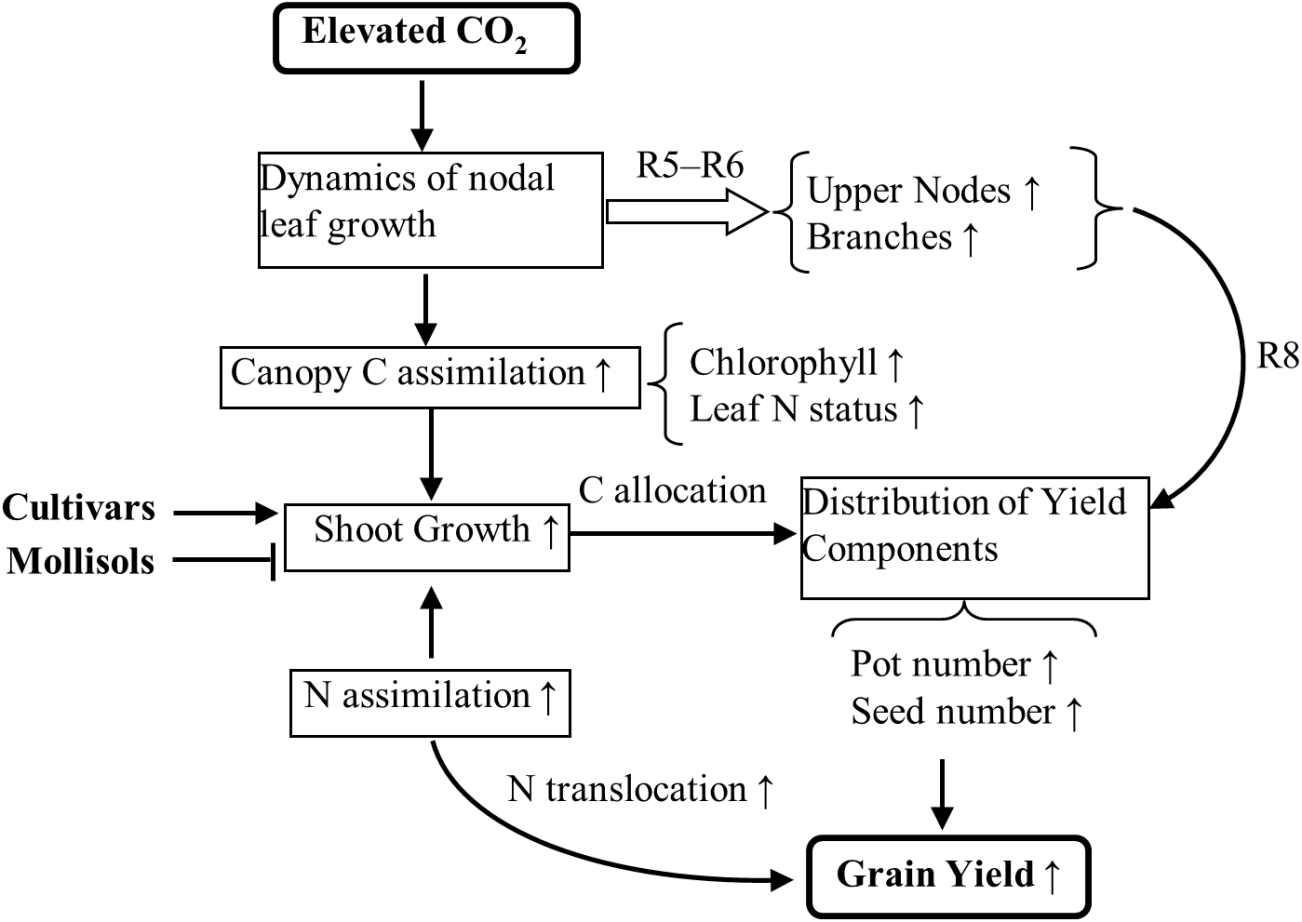
Diagram illustrating the impact of elevated CO_2_ on the nodal leaf growth and N assimilation in relation to carbon (C) assimilation and yield response. Two soybean cultivars were grown in Mollisols with low and high soil organic C. Plants were exposed to 380 or 580 ppm of CO_2_ for the entire growth stage. Symbol “↑” indicates an increase and “┴” indicates no significant effect.

Fig 7 summarized the effect of eCO_2_ on grain yield in soybean. Elevated CO_2_ significantly altered the distribution of nodal leaf area with a significant increase of leaf area at the upper nodes and on branches, especially during the period from R5 to R6. Consequently, the number of pods and seeds at these correspondent positions markedly increased, contributing to the yield gain under eCO_2_. The increase in the number of nodes and branches in response to eCO_2_ created a larger sink for photosynthates and sites for additional seeds. The enlarged C sink under eCO_2_ also required greater amounts of N for uptake and translocation to seeds. Soybean cultivars varied in their yield responses to eCO_2_, but the yield responded similarly in the two Mollisols. Elevated CO_2_ did not impact grain quality with no change in protein concentration.
